# Treatment of Abortions

**Published:** 1888-04

**Authors:** R. S. Gregg

**Affiliations:** Manor, Texas


### TREATMENT OF ABORTIONS.

*By R. S. Gregg, M. D., Manor, Texas.*

Read at Austin District Medical Society, March 8, 1888, and ordered published, by the Publishing Committee, in the Official Organ of the Society.

UPON examining the literature on frequency of abortions, we find it difficult to arrive at anything definite. Some authors even seem to think that abortions occur more often than labor at full term.

When we consider, then, this great frequency, and the many uterine diseases that may arise from neglect in treatment, it behooves us to be well prepared to treat the various forms o abortions that we, as physicians are compelled to meet. When called to a case supposed to be an abortion, or an attempt at an abortion, of course we must first establish our diagnosis. This is often by no means an easy matter, especially during the first two months of pregnancy. We consider it only necessary to mention a few of the most positive proofs of the existence of a threatened abortion. If we find all the usual signs of pregnancy and learn upon inquiry and by observation that the pains are intermittent, and that hemorrhage exists, and that this flow increases as the pains increase, and that pains are not relieved by free hemorrhage, we may be almost certain of a threatened abortion, although the internal os, may be so closed that we cannot feel the ovum or appendages upon digital examination. The diagnosis having been established our course of treatment should depend upon the advancement of the abortion. If we should find upon examination the os not very patulous, the life of our patient not endangered from excessive loss of blood, we must use every available means to prevent the abortion. Absolute rest must be insisted upon, elevation of the foot of the bed, cold acidulated drinks, and most important of all, we must hasten to bring our patient under the influence of opiates, to arrest uterine contractions. Ergot is sometimes used to arrest an abortion, but from the well known effects of this drug upon the uterus it would seem to be contra-indicated. Hypodermics of morphia and atropia will accomplish this most speedily. Sometimes we find a patient who, from some idiosyncrasy cannot take morphine, then we should resort to some opiate rectal suppositories. If, after our patient has been brought under the influence of the opiate, hemorrhage continues, and we find the os becoming more patulous, and can feel the ovum or membranes protruding into the cervix, it would be useless to longer attempt to arrest the abortion, and we should proceed to deliver. Nothing answers so well to accomplish this as the fingers. If the os is sufficiently dilated to allow us to feel any part of the uterine contents we can generally introduce one or two fingers of the right hand and by pushing down the fundus with the left hand, easily deliver. If the os will not admit our fingers we should then give ergot, and should hemorrhage be alarming, introduce a tampon. Often the ergot will be found sufficient. Sometimes the tonic contractions produced by the ergot will close the os rigidly, and we may seem farther from accomplishing a delivery than ever, but if it has arrested the hemorrhage, we should feel satisfied, for when the effect of the ergot has passed off, the os will generally be found sufficiently dilated to allow us to remove with ease the now thoroughly detached uterine contents. This may be accomplished by the fingers, forceps or dull curette. Should alarming hemorrhage begin after the delivery is complete, we must use hot intra-uterine injections, and continue the use of ergot. If the uterus is flabby, and os fully dilated, and we can obtain ice more readily than the hot water, apply it to hypogastrium, and insert a piece into the uterine cavity. Such means will generally control the hemorrhage. The question will now arise, shall we use antiseptic vaginal or intra-uterine injections ? If the abortion has been caused by any of the eruptive fevers, or from a high temperature from any cause, we should most certainly take precaution, but if it has followed some slight accident, over-exertion, nervous shock, or occurs in a woman, whom we know to abort habitually, there is no great need of such treatment.

We should always use antiseptic injections when the lochia is fœtid. Frequently we are not called for several days after the abortion has been in progress and will find this condition of the lochia. It may then be difficult to determine whether it is complete or partial. Of course the best means of ascertaining this is to introduce the fingers, and if this is not possible, and hemorrhage and pains continue, we should use the curette. Hemorrhage is not often so severe as we find it at full term from uterine inertia, or from an abnormal implantation of the placenta. We will often find, after an abortion, hemorrhage recurring every two or three weeks, especially when the woman takes unusual exercise. Such cases should be thoroughly examined for a portion of retained placenta, required to rest in bed, and put upon an iron and ergot treatment. Cannabis indica sometimes acts much better than ergot in such cases. It is often difficult to get our patients to recognize the importance of rest after an abortion, and from this fact so many women will date their uterine troubles to a former abortion. Subinvolutions, and displacements are frequent sequelæ of abortions.

No physician can follow his profession very long without encountering that class of abortions we usually term habitual. Of course there must be some cause for this unfortunate condition, and to treat it rationally, we should faithfully try to ferrit it out. If the cause can be ascertained and removed, we may expect to relieve our patient. Should we fail to find any satisfactory cause, we can often by appropriate treatment, tide our patient over her usual time for aborting, and thus relieve her. These abortions will generally be found to occur at a time when the menses would have appeared had the patient not conceived. Rest in bed at this time, and so long as there are any symptoms of an abortion, should be insisted upon. Some physicians highly recommend the continued use of the fluid extract viburnum prunifolium in such cases. My experience with this drug is sight. Morphia and bromide potassium combined will relieve the uterine of irritability, and quiet the contractions. It is not often necessary to continue this prescription long enough to endanger our patients with the morphine habit. Often the contractions will cease if the woman will assume the recumbent position, without the use of any medicine. Another important point is to warn our patients against constipation. We often find the rectum impacted with hard fœces, although our patient has assured us that her bowels act every day. This continued pressure on the gravid uterus may bring on the contractions, especially during the first two months of pregnancy. If we wish to carry the woman safely through to full term we must also insist upon a complete cessation of the marital relation during that pregnancy, since coitus cannot fail to excite an already irritable uterus.

